# Food Inspection

**Published:** 1892-11

**Authors:** Olaf Schwarzkopf


### FOOD INSPECTION.*

By Olaf Schwarzkopf.

In looking over the veterinary and medical journals and the daily press of the country during the last year, it must have become apparent to anyone that a great change in sentiment has taken place in regard to meat and milk inspection, not only in professional medical men, but also in the great public. A few years ago the matter was hardly discussed anywhere, whereas to-day it is an important issue in many states and cities, and its practical application is more or less considered all over the country. Still, the practical side of the question is yet almost entirely neglected. Judging from some occurrences of the last year, much patience and prudence will be required by veterinarians and other experts in properly establishing this branch of sanitary science as needed.

The first matter I shall have to explain before the Association is the dispute which has arisen between Dr. W. L. Williams of Indiana, and myself on actinomycosis bovis in its relation to meat inspection. Dr. Williams, as chairman of this committee for 1891, brought forth a lengthy report at the last meeting in Washington, in which he severely criticised portions of my original paper on national and international meat inspection, especially the stand I had taken on actinomycosis. I claimed at the time that meat from cattle so diseased might be fit for human food in parts, according to examination, giving no reasons for such position, because the character of my paper did not permit me to do so. Dr. Williams, in his report, took the opposite stand, arguing that actinomycosis is a contagious disease and the meat dangerous as food; he explained his position at length, intermixing it freely with a goodly supply of unfriendly compliments for me. I should have been glad to have the matter lie over until to-day, as Dr. Williams thinks I ought to have done; but the great timely interest attached to the question, together with his peculiar, aggressive style, compelled me to reply to his report before this meeting in our professional journals. In this reply I confined myself to compiling facts which went to show that actinomycosis cannot be considered a contagious disease, and that the meat of animals affected with a localized actinomycotic tumor is looked upon as innocuous by our authorities on actinomycosis. To this again Dr. Williams replied in our veterinary journals, trying hard theoretically and inferentially to sustain

* Report of the Special Committee on Food Inspection at the 29th Annual Meeting of the United States Veterinary Medical Association.

his views of the contagious character of the disease. I gladly admit that some of his arguments are forcible, and have made me still more cautious than I naturally am; but the general strain of his ideas is without scientific accuracy, and seems to indicate that he is gradually shifting around to another view. This, at least, may be inferred from the conclusion of his latest article which reads about as follows: "It seems that Dr. Schwarzkopf would have the reader believe that the committee held in the report that actinomycosis was highly contagious; that it was generally due in animals to transmission from animal to animal; that it could be readily transmitted from animal to animal or man; and that the destruction of the meat from animals mildly affected was an advisable sanitary measure. A careful reading of the text of the report will clearly show that those inferences have no foundation." I am unable to extract any other view out of the general tenor of Dr. Williams' articles than mentioned, and take this utterance for an unconscious retreat. But I hope that he will soon become conscious of the fact that he has been fighting on the wrong side, not more for his own sake than for the conversion of those American veterinarians, who, less gifted and learned than he, have been blind followers of his views.

In the meantime I shall continue to sustain my standpoint on actinomycosis, and I take pleasure in reading to you a letter from Prof. Mayo of the Kansas Agricultural College and Experiment Station, which he kindly consents to have embodied in my report. He writes under date of August 24th inst.

"PROF. SCHWARZKOPF,

"MINNEAPOLIS, Minn.

"Dear sir:

"Pursuant to mine of the 22nd, I send you a brief report of my work upon actinomycosis bovis, or that part of it relating more particularly to meat inspection.

"I have been at work at this disease during the past three years, and the more work I do with this disease, the more firmly am I convinced that the essential part of the life history of this organism is yet to be worked out. inspectors and others to the contrary notwithstanding.

"In many autopsies held upon 'lump-jaws,' where specimens have been collected and examined microscopically, I have never found an animal affected in any other place—unless purposely inoculated—than evident upon an ocular examination. The little fibrous tumors found along the intestines and in the mesentery (see Rept. Ill. Live-stock Comm., 1890, p. 17), are not actinomycotic in nature, and have nothing to do with the disease.

"Of fifty inoculations made upon animals, thirty-eight were made with pus, most of them taken directly from a cavity and transferred to point of inoculation immediately before it could be cooled to any extent, a few well inoculated with pus allowed to dry from two hours to two weeks. I have used it in sufficient quantities to form an abscess. I have also produced an abscess artificially, and inoculated it with fresh actinomycotic pus. I have inoculated in the tongue, jaw, neck, shoulders, etc. I have fed animals oats soaked in warm water in which a large amount of pus had been stirred, first wounding the tongue or mucous membrane of jaws and cheek. All of the thirty-eight inoculations were *total failures.*

"Of twelve inoculations made with a neoplastic growth which formed after an operation on an actinomycotic tumor, five were successful; yet I can hardly call it an inoculation. It is a transplanting of the disease

"I have also tried growing the organism on agar-agar, plain and nutrient, on gelatine, bouillon and blood serum, surface cultures and anærobic, at room temperature and in an incubator, and though the organism will remain intact and of normal appearance for months, yet upon a careful examination I have failed to discover any growth of the organism itself.

"I do not think this disease can be conveyed from one animal to another except by direct transplantation, and I am of the opinion that we must look for the source of infection outside of the animal body. I do not think the animal economy is a natural place of abode of this parasite, and the conditions in the animal economy are not sufficiently favorable for the organism to mature its spores to a stage where they will germinate under favorable conditions, hence the 'rosettes' as found in the pus are harmless, because incapable of growth.

"I consider the flesh of actinomycotic animals as perfectly harmless as food. I am of the opinion that they ought to be condemned only when general emaciation indicates mal-nutrition, not because they are affected with actinomycosis.

"These opinions have been formed as the result of my own work When I commenced work, through the influence of my former teachers, I considered it a 'dangerously contagious' disease, and the flesh as unfit for food.

"I am firm in my opinions, but not rabid, and am open to conviction to a contrary opinion when careful and truthful work shall bring forward evidence that does not now exist, which will show this disease to be of a 'dangerously contagious' nature.

"Yours, etc."

I lay a good deal of weight upon this letter because the observations come from an American veterinarian who has worked independently of foreign investigators, and who was under the influence of adverse teaching.

Other investigations about actinomycosis have been made by Profs. Max Wolf and James Israel published in Virchow's Archives, vol. 126, I. They report the successful cultivation and inoculation of actinomyces which were taken from two cases of human actinomycosis. They succeeded in cultivating it on agar-agar at 37 degrees Celsius, and under anærobic conditions. The microscopical examination of these pure cultures showed micro-organisms of very different form and size; short and long rods, which were solid or jointed; filaments with and without bifurcation, some of spiral forms, others resembling cocci. The club form, so characteristic of actinomycosis bovis, did not appear in the culture. From these results the investigators classify the micro-organisms with the bacteria.

Intraperitoneal inoculation on eighteen rabbits and three guinea pigs with pure cultures resulted in producing tumors of the size of a bean or hazel nut in which the clubs could be found. The inoculation of one sheep had a negative result; experiments with calves or cows were not made.

Still another discovery of interest is reported in the "Lancet" of July 2, 1892. As early as 1886 Dr. Vandyke Carter suggested the identity of the Indian disease, commonly known as Madura foot with the European form of human actinomycosis. Recently Prof. Crookshank received a specimen of Madura foot, and Mr. Hewlett, under his direction, examined it by the modern methods used for actinomycosis, and reports as follows: The disease occupied the whole of the foot and the lower third of the leg. The foot was enlarged, the bones softened and small abscess cavities were scattered through the tissues and communicated with the surface, the external openings of which had raised and thickened margins. Specimens of these were stained by Gram's method, and showed a fine network of branching and interlacing filaments stained blue, but the club-shaped bodies were apparently absent. By staining with orange-rubin, however, clubs can be demonstrated at the periphery. The microscopical appearances of Madura disease, both in the stained and unstained conditions, are identical with those of actinomycosis; clinically and pathologically also the diseases resemble each other closely, and there can no longer be any doubt in pronouncing Madura foot a manifestation of actinomycosis in man.

As nothing is said in Mr. Hewlett's report about the etiology and symptoms, and as the disease was unknown to me, I consulted "The Reference Handbook of the Medical Sciences," by C. Buck, M.D., where following is said: "The disease is always protracted in duration; four, six or even twenty or more years may elapse before the foot becomes so entirely disorganized as to demand amputation. The affected foot may, in some cases, exceed its fellow four or five times in bulk, and obtain a weight of a dozen pounds or more.

"The origin of the disease is commonly attributed to the entrance of a thorn or splinter, or to a bruise inflicted upon the parts."

No one in India, therefore, has ever pronounced the disease contagious.

The curative treatment of actinomycosis has been generally confined to surgical measures and to the application of iodine, carbolic acid, caustics, etc. In this way starting tumors have readily yielded to treatment, whereas tumors already affecting bones and actinomycotic glossitis, were looked upon as hopeless cases. The news, therefore, will be welcomed that Prof. Thomassen, of the Utrecht Veterinary School, pronounces iodide of potassium as a specific in every form of the disease when given as a drug. Prof. Nocard, at a recent meeting of the Paris Central Veterinary Society, in giving an instructive account of the disease, fully sustains this report from his own experiments. He gives the drug daily in doses of six grammes (about 3½ drams) in a pint of water, until signs of iodism appear, or until improvement commences, which may be usually expected within eight days. Then the dose is reduced to five or four grammes (about 2½–2 drams). The animals endure this treatment well, and in less than eight days can masticate hay; from eighty cases a cure has generally been effected, and on an average, in fifteen days.

To my mind this discovery of our esteemed French and Holland colleagues is very valuable, and more scientific and humane than the advice of the Illinois Live-stock Commission and some teachers of the Chicago Veterinary College, viz: The wholesale condemnation and destruction of cattle so diseased, or, not to forget the pledge of the members of the Northeastern Iowa Veterinary Society to discountenance the removal of actinomycotic tumors, thereby enabling the owner of such animals to dispose of them to unsuspecting parties.

Turning now to a short consideration of the practical application of meat inspection in this country, I am glad to report that a number of larger cities have established Bureaus of Meat Inspection, employing one or several veterinarians as meat inspectors, instead of the old custom of employing butchers, or—what is still worse—political impostors. However, that such a desirable institution is not always and easily and quietly obtained, was shown by the recent struggle in Buffalo, N. Y. The Health Commissioner of that city is a progressive man, and was of the opinion that the office of cattle inspector should be filled by a well educated and practically informed veterinary surgeon. Under the civil service law, however, he is required to confine his appointments to such applicants as are certified to be competent by the examining committee. Here it transpired—incredible as it may seem—that from three veterinarians and two butchers, which formed the eligible list, a butcher came out at the head of the ticket with 90 per cent. and marked "veteran." To this the Health Commissioner objected, as in his opinion the butcher's paper could only be marked 45 per cent., although he admitted that the veterinarian was also marked too high.

Judging from newspaper slips which were forwarded to me, the controversy must have been warm and interesting, some civil service commissioners maintaining the view that a butcher was as competent for the position as a graduate of a veterinary college; and in regard to bad spelling and incorrect use of technical words, they said, it was shown by the examination that none of the veterinarians could have passed a satisfactory examination.

I must confess that the answers of one veterinarian, as printed in full in the *Buffalo Courier*, were amazing in regard to illiteracy and lack of professional knowledge. I am really ashamed to reproduce them before this Association and do not wish to dwell any longer on the subject except to say that if such public examples should become common they will badly reflect on our veterinary colleges. The outcome of the contest in Buffalo was, I understand, a new examination and the ultimate employment of a veterinary surgeon.

The condition of meat-inspection in Europe cannot be uniformly judged, as there is too great a variance in different countries. England—generally speaking—seems to be not better off than our own country, while it cannot boast of a national law as we now have in effective operation in regards to international inspection. Austria, Spain and Russia have made little attempts at inspection, although St. Petersburg has a very fine public abattoir which possesses the only museum for meat inspection in existence. Germany, France and Italy, however, are undoubtedly those countries where meat-inspection is most highly developed, and the new national law of Italy, enacted a year or two ago, is considered by many the most perfect inspection law in enforcement. I have been unable to collect facts about meat inspection from France and Italy, but I wish to report some interesting data from Germany, gathered from some of her veterinary journals.

Prof. Hirschberg, of Berlin, announced before the Berlin Medical Society that he had examined—within the sixteen years between 1869-1885—60,000 people with diseases of the eye, of which seventy cases were traced to the lodgment of a cysticercus within the eye. During the last six years he had examined 46,000 people and he has found the parasite only in two cases, of which one was from Saxony. He says that this fact cannot be merely incidental, but must be looked upon as a triumph of meat inspection at Berlin. Prof. Virchow sustained this view from the observations in his clinic in regard to cysticerci in the brain. The proportion of 1:31 had lessened within the last eight years to 1:280.

But even the domestic dog seems to benefit the meat inspection in Berlin. Dr. Deffke, in his article on the "Entozoa of the dog," states that animal parasites of the dog have greatly diminished at Berlin. The older sick-reports of the Berlin veterinary hospitals show that the treatment of taeniæ has been quite common, whereas it is rather seldom at present. The taenia marginata of the dog, for instance, from which the cysticercus tenuicollis of slaughter animals develops, was found by Deffke only in 7 per cent. of post mortem examinations, while Schone, in Saxony, found them in 27 per cent. and Krabbe, in Iceland, in 75 per cent.

Since game has been included in the meat inspection in Germany a wild boar at Berlin has been found diseased with trichinæ and several species of river-fishes have been found to be affected with cysticerci of the meat. The so-called actinomyces musculerum sius, discovered by Dunker, has also been found by him in the muscles of sheep; the meat from such animals had a watery appearance.

An invention of great importance for public slaughter-houses evidently is the Kafill-Disinfector. The original apparatus was invented by De la Croix, a veterinarian at Antwerp, but it has undergone some improvements. This apparatus is now in use in many abattoirs in Germany, Holland, Belgium, etc., and is highly recommended as a substitute for rendering tanks or other apparatus employed for the destruction and utilization of condemned carcases. It consists of a sterilizing and drying apparatus of large dimensions, reducing animal matter into fat, glue and powdered fertilizers. This disinfector works now to perfection and absolutely without any bad odor, which seems to be unavoidable in the use of crematories.

Another apparatus of interest is that of Rohrbeck, with which experiments have been made at the Berlin abattoir. It is also a sterilizing apparatus, but not for the destruction but the preservation of meat. In this apparatus large pieces of meat can be heated to a degree which not only destroys all animal parasites, but also all kinds of bacteria and germs of organic nature, without reducing the nutritive value of meat or altering its natural taste. Should the question be settled that meat so prepared can be safely sold for food, the disinfector of Rohrbeck would soon be in general use at the public abattoirs of Europe.

---

Whether milk inspection is really anywhere properly enforced in this country I have been unable to find out in time for this report. The States of Massachusetts, New York, New Jersey, New Hampshire, Pennsylvania, Connecticut, Ohio, Indiana, Wisconsin, Minnesota and Iowa have so-called dairy and food commissions, and many other States have dairy and food laws that are enforced by the boards of health of those States. In addressing the dairy commissioners and secretaries of States, I have secured a number of reports which are interesting reading matter for a veterinarian. But a glance at them shows that they are established on a commercial basis for the assistance and enlightenment of farmers and dairymen. The chemist is the only professional man whose work is called for by these commissions; he fills the pages of the reports with analyses of milks and gives useful instruction as to the best methods for butter and cheese factories. It is certainly important that we consume milk, butter and cheese, the composition of which is natural and pure, and we should not underrate the services of chemists in such places as hygienists. But it is equally true that a chemical analysis of milk does not reveal the presence of tubercle-baccilli or other germs with which milk is so abundantly infected, constituting, as is well known, by far the gravest danger to the human consumer. We must go back to the stables and cows that produce such milk; and here, as well as in the laboratory, comes in the value of the veterinarian. This fact is apparently not sufficiently appreciated by the dairy commissions, or they have not yet seen fit as yet, from political reasons, to include the services of a veterinarian in this work. Occasionally I find a report from so called inspectors about the hygienic condition of stables and dairy stock, but these reports give evidence that they cannot possibly come from a qualified veterinarian, although some are signed V. S.

I think we should give the matter of milk inspection our earnest consideration, for there is a great field of research before us and a most valuable public service in another direction of hygiene. Attempts should soon be made to secure such positions on the staff of these dairy and food commissions, and make them what they ought to be, and another upward step will be taken by the veterinary profession of this country.

